# Neuroimmune Mechanisms in Equine Asthma: Primary Inflammatory Triggers, Neuroimmune Modulation and Chronic Airway Remodelling

**DOI:** 10.3390/ani16121832

**Published:** 2026-06-14

**Authors:** Małgorzata Wierzbicka, Aleksandra Samsel, Marta Siemieniuch-Tartanus

**Affiliations:** Department of Large Animal Diseases with the Clinic, Institute of Veterinary Medicine, Warsaw University of Life Sciences, Nowoursynowska 100, 02-787 Warsaw, Poland; malgorzata_wierzbicka@sggw.edu.pl (M.W.); aleksandra_samsel@sggw.edu.pl (A.S.)

**Keywords:** equine asthma, neuroimmune interactions, neurogenic inflammation, substance P, neurokinins, airway innervation, neuronal remodelling, TRPV1, horse, recurrent airway obstruction

## Abstract

Equine asthma is a chronic inflammatory disease of the lower respiratory tract, primarily associated with inhalation of organic dust, microbial particles and environmental aeroantigens. Although the inflammatory and immunological mechanisms underlying equine asthma have been extensively investigated, the potential contribution of neuroimmune pathways remains poorly understood. In humans and rodent models, asthma is increasingly recognised as a disorder involving complex bidirectional interactions between the nervous and immune systems. Sensory nerve activation, neuropeptide release, autonomic dysregulation and neuronal remodelling contribute to bronchoconstriction, airway hyperresponsiveness, mucus hypersecretion and chronic airway remodelling. This review summarises current knowledge of the neuroimmune mechanisms involved in asthma, with particular emphasis on comparative aspects across humans, rodents and horses. Literature searches were conducted using the PubMed database, focusing on studies investigating neurogenic inflammation, airway innervation, neuropeptides, transient receptor potential channels and neuronal remodelling in asthma and chronic airway disease. Existing equine evidence indicates the presence of substance P- and calcitonin gene-related peptide-immunoreactive nerve fibres in the equine airways, increased neurokinin-mediated bronchoconstriction in severe equine asthma, and enhanced airway innervation in affected horses. However, compared with human and rodent studies, horse-specific data remain extremely limited. Current evidence suggests that neuroimmune pathways are unlikely to be the primary initiating mechanism of equine asthma, but may act as important modulators of chronic airway dysfunction and disease progression. The marked scarcity of equine studies investigating neuroimmune signalling represents a major knowledge gap and highlights an important direction for future research in equine respiratory medicine.

## Simple Summary

Equine asthma is a chronic inflammatory disease of the lower airways, primarily triggered by exposure to organic dust, fungal particles and other environmental irritants. Although inflammation and immune dysfunction are considered the main drivers of disease, increasing evidence from human and experimental asthma research indicates that the nervous system also plays an important role in airway pathology. Interactions between sensory nerves and immune cells may contribute to bronchoconstriction, cough, mucus hypersecretion and chronic airway remodelling. This review summarises current knowledge of neuroimmune mechanisms involved in asthma across species, with particular emphasis on horses. Existing equine studies demonstrate increased airway innervation, neurokinin-mediated bronchoconstriction and the presence of neuropeptide-containing nerve fibres within the respiratory tract. However, many neuroimmune pathways described in humans and rodent models remain poorly investigated in horses. A better understanding of neuroimmune signalling in equine asthma may improve understanding of chronic disease progression and help identify novel therapeutic targets for severe equine asthma.

## 1. Introduction

Equine asthma is a chronic, non-infectious inflammatory disease of the lower airways, characterised by airway hyperresponsiveness, bronchoconstriction, mucus accumulation and impaired pulmonary function [1,2,3]. The terminology used to describe equine chronic inflammatory airway disease has evolved considerably over the past decades. Earlier descriptions referred to the condition as chronic obstructive pulmonary disease (COPD) because of perceived similarities to human COPD. Subsequently, the terms inflammatory airway disease (IAD) and recurrent airway obstruction (RAO) were introduced to distinguish milder and severe clinical presentations. More recently, an international consensus statement recommended the umbrella term “equine asthma” to encompass both mild-to-moderate equine asthma (MEA) and severe equine asthma (SEA), reflecting the shared pathophysiological features of these syndromes [4]. Environmental exposure to organic dust, fungal particles, endotoxins and other airborne contaminants derived from hay and bedding material is considered the principal initiating factor in the development and exacerbation of the disease [5,6,7].

Current understanding of equine asthma concentrates on inflammatory and immunological mechanisms, primarily neutrophilic airway inflammation, epithelial dysfunction, oxidative stress, and airway remodelling [8,9,10]. However, mounting evidence from human medicine and experimental rodent models suggests that asthma should not be viewed solely as an inflammatory disorder, but rather as a disease involving extensive interactions between the immune and nervous systems [11,12,13,14]. Neuroimmune signalling pathways contribute to airway smooth muscle contraction, sensitisation of the cough reflex, mucus secretion and chronic airway remodelling through bidirectional communication among sensory nerves, immune cells and structural airway cells [13,15].

In human asthma, neurogenic inflammation mediated by sensory nerves and neuropeptides such as substance P (SP), neurokinin A (NKA) and calcitonin gene-related peptide (CGRP) has been extensively investigated. Increased expression of substance P and neurokinin receptors has been demonstrated in airways of asthmatic patients, while sensory nerve remodelling and enhanced neuroimmune signalling have been linked to airway hyperresponsiveness and chronic inflammation [13,16]. Similarly, rodent models have demonstrated substantial involvement of neuronal plasticity, neurotrophins and transient receptor potential channels in chronic airway disease [12,13,15]. In contrast, the role of neuroimmune pathways in equine asthma remains poorly characterised, despite emerging evidence of increased airway innervation and altered tachykinin signalling in horses with SEA [17,18]. Importantly, neuroimmune mechanisms in equine asthma should not be interpreted as the primary initiating cause of disease. Inhaled environmental particulates and organic dust remain the principal drivers of airway inflammation in horses [5,6,7]. Neuroimmune pathways may act as important secondary modulators that amplify bronchoconstriction, airway hyperresponsiveness, cough reflex sensitivity and airway remodelling, thereby contributing to chronic disease progression and persistence of clinical signs in horses. Experimental studies in humans and rodent models have demonstrated that sensory nerve activation and neuronal remodelling enhance airway hyperreactivity and chronic airway dysfunction [13,15,19], while equine studies indicate increased neurokinin-mediated bronchospasm and enhanced airway innervation in severe equine asthma [17,18].

Therefore, the objective of this review was to summarise current knowledge of neuroimmune mechanisms implicated in asthma and chronic airway inflammation, with particular emphasis on comparative aspects across humans, rodent models, and horses. Additionally, this review aims to identify major gaps in current knowledge regarding equine neuroimmune respiratory pathology and to highlight potential directions for future research.

## 2. Materials and Methods

Literature Search Strategy

This narrative review was conducted using a structured literature search focused on neuroimmune mechanisms involved in asthma, with particular emphasis on equine asthma and comparative evidence from humans and rodent models. The primary database used was PubMed. Additional cross-referencing of relevant articles was performed using citation tracking from selected publications.

The literature search was conducted between January and May 2026. Searches included publications written in English and published between 1980 and 2026.

Search Terms

The following combinations of keywords and Medical Subject Headings (MeSH) terms were used:

- equine asthma AND neuroimmune
- equine asthma AND nervous system
- equine asthma AND substance P
- equine asthma AND neurokinin
- equine asthma AND CGRP
- equine asthma AND airway innervation
- recurrent airway obstruction AND neuropeptides
- horse asthma AND TRPV1
- airway neurogenic inflammation AND asthma
- sensory nerves AND asthma
- tachykinins AND asthma
- substance P AND airway inflammation
- TRPV1 AND asthma
- neuroimmune interactions AND asthma
- neuronal remodelling AND asthma
- BDNF AND airway smooth muscle
- NGF AND asthma
- pulmonary neuroendocrine cells AND asthma

Search Results and Study Selection

The initial PubMed search yielded approximately:

- 1 record for equine asthma AND neuroimmune
- 2 records for equine asthma AND substance P
- 3 records for equine asthma AND neurokinin
- 3 records for equine asthma AND airway innervation
- 366 records for substance P AND airway inflammation
- 208 records for TRPV1 AND asthma
- 190 records for neuroimmune interactions AND asthma
- 86 records for neuronal remodelling AND asthma
- 94 records for pulmonary neuroendocrine cells AND asthma

Titles and abstracts were screened manually for relevance. Studies were included if they:

1. Investigated neuroimmune or neurogenic mechanisms underlying asthma or chronic airway inflammation;
2. Examined neuropeptides, airway innervation, autonomic signalling, TRP channels, or neuronal remodelling;
3. Included equine, human or rodent asthma models relevant to comparative respiratory pathophysiology;
4. Were original research articles, consensus statements or high-quality review papers.

Studies were excluded if they:

1. Were unrelated to respiratory disease or asthma;
2. Focused exclusively on non-respiratory neurological disorders;
3. Were conference abstracts without full peer-reviewed publication.

Particular emphasis was placed on horse-specific evidence regarding airway innervation, tachykinin signalling, and neurogenic inflammation, given the limited number of equine studies that directly investigate neuroimmune mechanisms. Comparative data from human asthma and murine models were used to contextualise the currently available equine findings.

**Figure 1:**
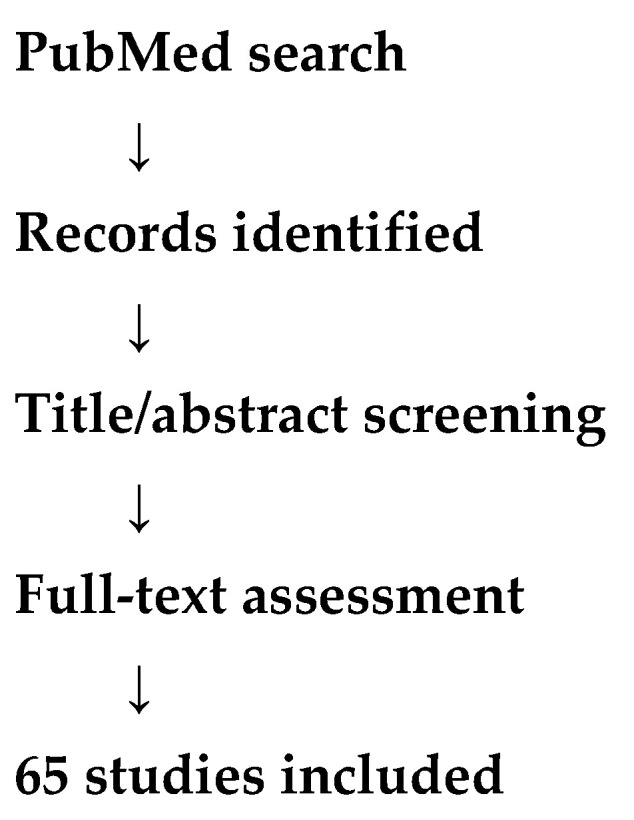
Data Extraction and Narrative Synthesis.

Following title and abstract screening, approximately 65 publications were retained for full-text evaluation. The final narrative synthesis included 27 equine studies, 15 human studies, 15 rodent studies, and 8 multispecies or studies describing mechanisms and pathways (Figure 1).

Given the limited number of equine-specific studies and the heterogeneity of methodologies among available publications, a systematic review or meta-analysis was not considered appropriate. Therefore, a narrative review approach was selected to integrate current mechanistic knowledge and identify gaps requiring future investigation.

Limitations of the Available Evidence

The present review was conducted as a narrative synthesis and therefore did not include a formal assessment of study quality or risk of bias. Nevertheless, the methodological characteristics of the available literature were considered when interpreting the findings. Most equine studies investigating neuroimmune mechanisms were based on relatively small sample sizes, cross-sectional designs, histological analyses, or ex vivo functional experiments. Furthermore, direct mechanistic investigations of neuronal signalling pathways in horses remain scarce, and many concepts discussed in this review are derived from human studies and experimental animal models. Consequently, conclusions regarding neuroimmune mechanisms in equine asthma should be interpreted with caution and treated as preliminary until confirmed by larger, more comprehensive equine studies.

A limitation of the present review is that the literature search was primarily based on PubMed. Although citation tracking was performed, relevant publications indexed exclusively in other databases may not have been identified.

## 3. Primary Initiating Mechanisms of Equine Asthma

Equine asthma is recognised as a chronic, environmentally induced inflammatory disease of the lower respiratory tract, primarily associated with inhalation of respirable organic dust, microbial contaminants, fungal spores, endotoxins and other airborne particulates derived from hay and bedding materials [5,7,20]. Repeated exposure to poorly ventilated stable environments rich in organic particulates is considered a major initiating factor for lower airway inflammation in susceptible horses [7,21]. Unlike classical allergic eosinophilic asthma in humans, SEA is predominantly characterised by neutrophilic airway inflammation, increased bronchoalveolar neutrophil proportions, and dysregulated innate immune responses, including cytokine imbalance and Toll-like receptor signalling [22,23,24,25]. Inhaled organic dust comprises complex mixtures of fungal particles, β-glucans, bacterial endotoxins and lipopolysaccharides that can activate airway epithelial cells, alveolar macrophages, and neutrophils [5,9,26]. Airway epithelial cells constitute the first immunological barrier to inhaled particles and actively participate in inflammatory signalling by secreting cytokines, chemokines and alarmins [27,28]. Activation of pattern-recognition receptors, including Toll-like receptors (TLRs), contributes to neutrophil recruitment and the amplification of inflammatory cascades in the airways by inducing cytokine and chemokine production by airway epithelial cells and innate immune cells [24,29,30]. In SEA, repeated exposure to environmental antigens leads to chronic inflammation, tracheal mucus accumulation, airway hyperresponsiveness and progressive structural remodelling of the lower airways [8,9,10].

Although adaptive immune responses also contribute to disease pathogenesis, equine asthma differs from classical human allergic asthma in its inconsistent association with IgE-mediated hypersensitivity and its predominance of neutrophilic rather than eosinophilic inflammation [22,25,26,31]. Nevertheless, equine asthma shares several pathophysiological similarities with human asthma, including airway hyperresponsiveness, reversible bronchoconstriction and chronic airway remodelling characterised by airway smooth muscle hypertrophy and structural alterations of the bronchial wall [8,22,32]. Consequently, mechanisms beyond conventional inflammation are increasingly investigated as contributors to disease chronicity and clinical severity.

Unlike human allergic asthma, equine asthma is characterised predominantly by neutrophilic airway inflammation [4,22,23]. Although interactions between eosinophils and sensory nerves are well documented in human and murine asthma, considerably less is known regarding neuroimmune interactions involving neutrophils [13]. Activated neutrophils release reactive oxygen species, proteases and cytokines that may influence neuronal excitability and airway sensory function [33,34]. Whether similar neutrophil–nerve interactions contribute to neuronal remodelling or airway hyperresponsiveness in horses remains unknown and warrants investigation.

## 4. Neuroimmune Signalling as a Secondary Modulator of Airway Dysfunction

**Figure 2:**
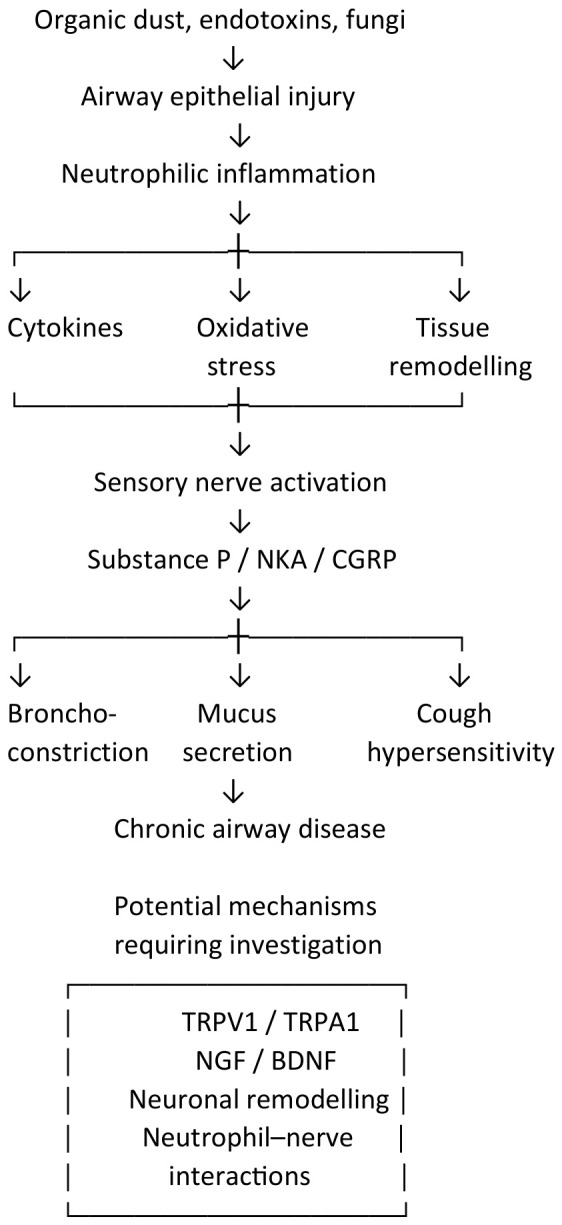
Proposed neuroimmune pathways in equine asthma.Legend: Solid lines = mechanisms supported by equine studies. Dashed lines = mechanisms inferred from human and rodent studies and requiring validation in horses.

Although inhalation of organic dust and microbial particulates remains the principal initiating factor in equine asthma, accumulating evidence from human and experimental studies suggests that neuroimmune pathways may substantially modulate airway dysfunction, bronchoconstriction, cough reflex sensitivity and chronic inflammation [13,17,18,19,35]. Rather than being the primary cause of disease, the nervous system likely acts as an amplifying and perpetuating component of airway pathology through sensory nerve activation, neuropeptide release and neuronal remodelling [13,15,18,35]. Proposed neuroimmune pathways in equine asthma are depicted in Figure 2.

Evidence suggests that several asthma-related clinical manifestations, including bronchoconstriction, mucus hypersecretion and cough, may arise from stimulation of unmyelinated C-fibre afferent nerves within the respiratory tract [19,36,37]. These fibres are activated by inflammatory mediators, allergens, particulate matter, temperature changes and tissue injury. Direct evidence regarding the distribution and functional properties of airway C-fibre afferents in horses is currently lacking. Their potential involvement in equine asthma is inferred primarily from anatomical studies that demonstrate substance P- and CGRP-containing nerve fibres within equine airways, and from functional studies of tachykinin signalling in horses. Activation of sensory nerve endings promotes the release of tachykinins and other neuropeptides, including SP, NKA and CGRP, which contribute to airway smooth muscle contraction, vasodilation, plasma extravasation and mucus hypersecretion, thereby amplifying neurogenic inflammation within the respiratory tract [38,39]. NKA is released predominantly from sensory nerve endings. Its principal physiological function is regulating smooth muscle contractility, particularly in the respiratory and gastrointestinal tracts. Dysregulated NKA signalling has been linked to exaggerated bronchomotor responses and airway hyperresponsiveness in inflammatory airway diseases [17,40]. In humans, tachykinins released from sensory nerves are important mediators of airway hyperresponsiveness and neurogenic inflammation. NKA is considered one of the most potent endogenous bronchoconstrictors in the lower respiratory tract, acting predominantly through NK-2 receptors expressed on airway smooth muscle cells [40,41]. SP is a member of the tachykinin family of neuropeptides and is widely distributed within sensory neurons innervating the respiratory tract. Under physiological conditions, SP contributes to nociceptive signalling, local vascular regulation and communication between the nervous and immune systems. Increased SP production and signalling have been associated with chronic inflammatory disorders and with enhanced recruitment of inflammatory cells [42,43]. SP predominantly activates NK-1 receptors and contributes to vascular permeability, leukocyte recruitment and airway oedema [44,45]. Similar mechanisms have been extensively demonstrated in murine models of allergic airway disease, where activation of sensory nerves enhances inflammatory responses and airway hyperresponsiveness [13,35].

CGRP is a sensory neuropeptide frequently co-localised with SP in airway afferent neurons. Physiologically, CGRP regulates vascular tone and tissue perfusion and may contribute to local neuroimmune communication within the respiratory tract. CGRP-containing nerve fibres have been identified in equine airways, although their functional role in equine asthma remains unknown [46].

Neuroendocrine cells and neuroepithelial bodies, which contain neuropeptides and neurotransmitters, are distributed among ciliated respiratory epithelial cells and function as specialised airway sensory structures [12,47]. Branchfield et al. [12] demonstrated that pulmonary neuroendocrine cells in mice can initiate type-2 immune responses via neuropeptide signalling pathways, supporting the concept that the airway epithelium serves as an active neuroimmune interface rather than merely a physical barrier.

The majority of bronchial innervation originates from autonomic fibres of the vagus nerve [14]. Cholinergic pathways regulate airway smooth muscle tone, mucus secretion, vascular permeability and bronchial blood flow [48,49]. During airway inflammation, inflammatory mediators may stimulate bronchospasm and exaggerate vagally mediated reflex bronchoconstriction, thereby amplifying airway obstruction [50]. Dysregulation of autonomic signalling may therefore contribute to persistent airway hyperresponsiveness in chronic respiratory disease [51].

Neuroimmune communication in asthma is bidirectional. Sensory neurons release neuropeptides that influence the recruitment and activation of inflammatory cells, while immune cells simultaneously secrete cytokines, lipid mediators and growth factors that can alter neuronal excitability and receptor expression [13,50]. SP is produced not only by neurons but also by several immune cell populations, including lymphocytes, eosinophils, dendritic cells and macrophages [43,52,53]. These neuropeptides promote cytokine secretion, neutrophil chemotaxis, mast cell activation and lymphocyte proliferation, thereby amplifying airway inflammation [43,52].

Neurotrophins, including nerve growth factor (NGF) and brain-derived neurotrophic factor (BDNF), are essential regulators of neuronal survival, differentiation and plasticity. Increased concentrations of several neurotrophins have been reported in patients with allergic asthma and experimental models of airway inflammation [15,54].

BDNF, produced by neurons, airway epithelial cells, and bronchial smooth muscle cells, are believed to contribute to neuronal remodelling and airway hypersensitivity [55,56,57]. Elevated expression of NGF and BDNF has been demonstrated in asthmatic airways and in experimental models of allergic airway inflammation, suggesting a role for neurotrophins in airway remodelling and neuronal plasticity [56,57]. During inflammation, TNF-α enhances BDNF synthesis in bronchial smooth muscle cells and promotes extracellular matrix remodelling by inducing collagen deposition, fibronectin synthesis and matrix metalloproteinase activation [55]. Together, these mechanisms establish a direct relationship between neuroimmune activation and structural airway remodelling [13,55].

Transient receptor potential vanilloid 1 (TRPV1) is a non-selective cation channel expressed predominantly on sensory neurons. It functions as a molecular sensor for noxious heat, protons and chemical irritants, thereby contributing to protective airway reflexes [58,59]. The transient receptor potential cation channel subfamily V member 1 (TRPV1), also known as the capsaicin receptor and the vanilloid receptor 1, is activated by capsaicin, heat, and arachidonic acid metabolites [58]. Chronic airway inflammation may increase TRPV1 expression on sensory nerve fibres, thereby enhancing cough reflex sensitivity and bronchoconstriction [59,60]. TRPV1-mediated hypersensitivity is well documented in both human asthma and experimental rodent models, in which increased TRPV1 activity contributes to airway inflammation, cough hypersensitivity, and bronchial hyperresponsiveness, suggesting that TRPV1 may represent a promising therapeutic target for chronic airway disease [59,61].

Compared with humans and experimental rodent models, evidence regarding neuroimmune mechanisms in equine asthma remains limited (Table 1). Nevertheless, available data suggest that similar pathways may also contribute to disease pathogenesis in horses (Table 2). Anatomical studies identified SP- and CGRP-immunoreactive nerve fibres in equine airways, particularly beneath the epithelium and around blood vessels and airway glands [57]. These fibres are strategically positioned to detect inhaled irritants and modulate inflammatory responses within the respiratory tract [11]. Sonea et al. demonstrated SP-binding sites in equine airway tissues, particularly in tracheobronchial glands, airway epithelium and pulmonary vessels, with most binding sites corresponding to NK-1 receptors [62]. These findings suggest that SP may contribute to the regulation of vascular tone, airway secretion and inflammatory responses in the equine lung. Activation of intrapulmonary afferent nerves containing SP by inhaled allergens or other noxious stimuli may promote mucus hypersecretion and airway narrowing secondary to vascular congestion [62].

Functional evidence for neuroimmune involvement in equine asthma primarily derives from studies of horses with RAO, now classified as SEA. Venugopal et al. [17] demonstrated that NKA induces more pronounced bronchospasm in horses with SEA than in healthy controls. Increased NK-2 receptor expression was observed in the bronchial smooth muscle, airway epithelium and pulmonary vessels of affected horses, supporting the hypothesis that tachykinin signalling contributes to airway hyperresponsiveness and bronchoconstriction in equine asthma [17].

Confirmed Neuroimmune Mechanisms in Horses

Although the number of studies investigating neuroimmune mechanisms in equine asthma remains limited, several pathways have been directly demonstrated in horses. Anatomical studies have identified SP- and CGRP-immunoreactive nerve fibres within the equine respiratory tract, particularly beneath the airway epithelium and around blood vessels and airway glands [46]. Furthermore, SP binding sites, predominantly to NK-1 receptors, have been localised in tracheobronchial glands, airway epithelium and pulmonary vessels [62]. Functional evidence is provided by studies showing enhanced NKA-induced bronchoconstriction and increased NK-2 receptor expression in horses affected by SEA [17]. More recently, increased airway innervation within and adjacent to airway smooth muscle bundles has been documented in horses with SEA, suggesting neuronal remodelling associated with chronic disease [18]. Collectively, these findings support the involvement of tachykinin signalling and altered airway innervation in equine asthma, although the precise functional consequences remain incompletely understood.

Neuroimmune Mechanisms Inferred from Human and Rodent Studies

Several neuroimmune pathways that are well established in human asthma and experimental animal models have not yet been directly investigated in horses. These include the role of TRP ion channels, particularly TRPV1 and TRPA1; neurotrophin-mediated neuronal plasticity involving NGF and BDNF; eosinophil-driven sensory nerve remodelling; and neuroimmune interactions mediated by pulmonary neuroendocrine cells [12,13,59]. In humans and rodents, these mechanisms contribute to airway hyperresponsiveness, chronic cough, neurogenic inflammation and structural remodelling of the airways. Whether similar processes occur in equine asthma remains unknown. Given the predominance of neutrophilic rather than eosinophilic inflammation in horses, direct extrapolation from human and murine studies should be approached cautiously. Nevertheless, these pathways represent promising candidates for future investigation and may help explain persistent airway dysfunction in severe equine asthma.

A major limitation in translating neuroimmune mechanisms from human and rodent asthma to horses is the difference in dominant inflammatory phenotypes. Human allergic asthma and most experimental murine models are largely driven by type-2/eosinophilic inflammation, whereas severe equine asthma is predominantly neutrophilic [13,22,25]. This distinction is particularly important for mechanisms of neuronal plasticity because eosinophil–nerve interactions and eosinophil-driven sensory nerve growth have been demonstrated in human and murine asthma, but their relevance to horses remains uncertain [13,50]. In equine asthma, neuroimmune interactions may instead involve neutrophils, macrophages, epithelial cells and airway smooth muscle cells, although these pathways have not yet been directly investigated [23,24,26,29,30]. Similarly, receptor-level evidence differs substantially across species: TRPV1/TRPA1 channels, neurotrophin receptors and pulmonary neuroendocrine cell pathways are well characterised in humans and rodents, whereas in horses, current evidence is largely restricted to tachykinin signalling, NK-1/NK-2 receptor localisation and increased airway innervation [12,17,18,54,59,60,61,62]. Therefore, direct extrapolation from rodent models should be approached with caution.

## 5. Neuronal Remodelling and Chronic Disease Progression

This growing body of evidence from human and rodent studies indicates that chronic airway inflammation is associated with structural remodelling of airway neuronal networks [13,18], suggesting that neuronal plasticity may be an important component of the pathogenesis of chronic respiratory disease. Persistent exposure to inflammatory mediators may increase airway nerve density, alter sensory nerve phenotypes and enhance neuronal excitability [13,54]. These neuroplastic changes are believed to contribute to chronic cough, persistent airway hyperresponsiveness and the partial irreversibility of airway dysfunction [13,59].

In murine asthma models, eosinophils directly stimulate sensory nerve growth and branching, thereby promoting airway hyperresponsiveness and enhanced sensory reflex responses [13]. Neurotrophins, including NGF and BDNF, appear to play central roles in this process by enhancing neuronal survival and promoting sensory nerve plasticity [54,55,57]. Chronic neuroimmune activation may therefore create a self-perpetuating cycle in which airway inflammation promotes neuronal remodelling, while altered innervation further enhances airway dysfunction and inflammatory signalling [13,55].

Table 1 shows a comparative overview of confirmed neuroimmune mechanisms implicated in asthma in humans, rodent models and horses. 

Table 2 shows a comparative overview of the current state of knowledge regarding neuroimmune mechanisms implicated in asthma in humans, rodent models and horses. 

The strongest horse-specific evidence for neuronal remodelling in equine asthma was recently reported by Leduc et al. [18], who demonstrated increased airway innervation in horses with SEA. Leduc et al. demonstrated that the increase in airway innervation was particularly evident within and adjacent to airway smooth muscle bundles [18]. Horses with SEA showed significantly greater nerve density than healthy controls, supporting the hypothesis that neuronal remodelling preferentially affects airway compartments directly involved in bronchomotor regulation. The authors proposed that increased neural input to airway smooth muscle may contribute to persistent bronchoconstriction and airway hyperresponsiveness. However, direct correlations with pulmonary function variables or bronchoalveolar lavage cytology were not established and require further investigation [18]. Increased airway innervation may amplify bronchoconstriction, contribute to airway smooth muscle dysfunction and promote progression of airway remodelling in SEA [17,18]. These findings suggest that neuroimmune pathways may contribute primarily to chronic disease progression and maintenance rather than to the initial induction of disease [13,17,18]. In horses, prolonged exposure to inhaled organic dust initiates airway inflammation, whereas neuroimmune dysregulation may subsequently perpetuate bronchoconstriction, cough hypersensitivity, mucus hypersecretion and structural airway remodelling [4,8,10]. These mechanisms may help explain why some horses continue to exhibit airway hyperresponsiveness and recurrent clinical signs despite reduced environmental antigen exposure [9,63]. Despite recent advances, substantial knowledge gaps remain regarding the role of neuroimmune signalling in equine asthma. Very limited data are available on concentrations of substance P, CGRP, NGF or BDNF in bronchoalveolar lavage fluid or airway tissues of asthmatic horses, and only a small number of studies have investigated neuroimmune mechanisms directly in equine airways [17,18,64]. Similarly, expression of TRPV1 and other TRP ion channels has not yet been systematically investigated in equine respiratory disease, although studies in humans and rodent models strongly support their involvement in cough hypersensitivity, airway inflammation and neuronal sensitisation [59,61]. In addition, interactions among airway nerves, neutrophils, epithelial cells and airway smooth muscle remain poorly characterised in horses, despite growing evidence that neuroimmune signalling contributes to chronic airway dysfunction and structural remodelling in asthma [10,13]. Further investigation of these pathways may improve understanding of disease chronicity and facilitate the development of targeted therapeutic strategies for SEA. Future studies integrating histopathology, immunohistochemistry, transcriptomics and biomarker analysis in bronchoalveolar lavage fluid may clarify the extent to which neuronal remodelling contributes to chronic airway dysfunction and treatment resistance in horses [13,18]. A better understanding of neuroimmune mechanisms could ultimately identify novel therapeutic targets to modulate sensory nerve activation, neuropeptide signalling, and neuronal plasticity in equine asthma [14,64].

**Table 1:** Confirmed neuroimmune mechanisms involved in asthma across species.

Mechanism	Humans	Rodent Models	Horses	Main Functional Consequence	Key References
Activation of sensory C-fibre afferents	Confirmed	Confirmed	Indirect evidence	Bronchoconstriction, cough, mucus secretion	[11,36]
Substance P-mediated neurogenic inflammation	Confirmed	Confirmed	Confirmed anatomical evidence	Vasodilation, plasma extravasation, leukocyte recruitment	[44,62]
Neurokinin A (NKA)-induced bronchoconstriction	Confirmed	Confirmed	Confirmed	Airway smooth muscle contraction and airway hyperresponsiveness	[17,40]
NK-1 receptor expression in airways	Confirmed	Confirmed	Confirmed	Regulation of vascular permeability and airway secretion	[16,62]
NK-2 receptor upregulation in asthma	Confirmed	Confirmed	Confirmed in severe equine asthma	Enhanced bronchoconstriction	[17]
CGRP-positive airway innervation	Confirmed	Confirmed	Confirmed anatomical evidence	Neurogenic vasoregulation and inflammatory signalling	[62]
Neuroendocrine cell activation	Confirmed	Confirmed	Not investigated	Initiation of immune responses through neuropeptide signalling	[12]
Vagal/autonomic dysregulation	Confirmed	Confirmed	Suspected	Reflex bronchoconstriction and airway hyperreactivity	[14,48]
TRPV1 upregulation and hypersensitivity	Confirmed	Confirmed	Not investigated	Cough hypersensitivity and bronchoconstriction	[59,60,61]
Neuronal remodelling/increased airway innervation	Confirmed	Confirmed	Confirmed	Persistent airway hyperresponsiveness and chronic disease progression	[13,18]
Eosinophil-driven sensory nerve plasticity	Confirmed	Confirmed	Not demonstrated	Increased airway sensory nerve density	[13]
Neurotrophin involvement (NGF/BDNF)	Confirmed	Confirmed	Hypothetical/indirect	Neuronal survival, branching and airway remodelling	[15,56]
Substance P production by immune cells	Confirmed	Confirmed	Presumed but unstudied	Amplification of inflammatory signalling	[43,52,64]
Neuroimmune interaction contributing to airway remodelling	Confirmed	Confirmed	Suspected	Fibrosis, smooth muscle proliferation and chronic airflow limitation	[15,55,65]
Predominant inflammatory phenotype associated with neuroimmune pathways	Usually eosinophilic/type 2	Usually eosinophilic/type 2	Predominantly neutrophilic	Species-specific inflammatory response profile	[4,22,64]

**Table 2:** Current state of knowledge regarding neuroimmune mechanisms implicated in asthma in humans, rodent models and horses.

Neuroimmune Component	Humans	Rodent Models	Horses	Current Level of Evidence in Horses	Research Gap in Equine Asthma	Key References
Substance P expression	Well documented	Well documented	Anatomical evidence only	Low	Lack of BALF/tissue quantification studies	[16,62]
Neurokinin A signalling	Well-documented	Well-documented	Increased bronchial responsiveness demonstrated	Moderate	No mechanistic molecular studies	[17,40]
NK-1/NK-2 receptor expression	Confirmed	Confirmed	Partially confirmed	Moderate	Limited receptor distribution studies	[17,62]
CGRP-positive nerve fibres	Confirmed	Confirmed	Confirmed anatomically	Low	Unknown functional significance	[62]
Sensory C-fibre activation	Well-documented	Well-documented	Suspected	Low	No functional electrophysiological studies	[11,36]
TRPV1 involvement	Extensive evidence	Extensive evidence	Not investigated	Very low	Complete absence of equine studies	[59,60,61]
TRPA1/TRPM8 pathways	Increasing evidence	Increasing evidence	Not investigated	Very low	Complete absence of data	[35,61]
Neurotrophins (NGF/BDNF)	Well-documented	Well-documented	Hypothetical only	Very low	No equine biomarker studies	[15,56]
Neurogenic inflammation	Extensive evidence	Extensive evidence	Suggested indirectly	Low	Lack of direct mechanistic evidence	[11,44]
Autonomic dysregulation	Well-documented	Well-documented	Suspected	Low	No autonomic function studies	[14,48]
Neuronal remodelling	Confirmed	Confirmed	Recently demonstrated	Moderate	Mechanisms remain unknown	[13,18]
Immune cell–nerve interactions	Extensive evidence	Extensive evidence	Unknown	Very low	Virtually unexplored	[42,43]
Eosinophil–nerve interactions	Confirmed	Confirmed	Unclear due to neutrophilic phenotype	Very low	Unknown relevance in horses	[13]
Neutrophil–nerve interactions	Limited evidence	Limited evidence	Potentially important	Very low	Major unexplored field	[23,64]
Neuroimmune therapeutic targets	Emerging therapies	Experimental therapies	Not investigated	Very low	No translational equine studies	[14,61]

Although neuroimmune mechanisms are not currently targeted directly in equine asthma therapy, they may have important clinical implications. Persistent sensory nerve activation and neuronal remodelling could contribute to ongoing airway hyperresponsiveness despite environmental management and corticosteroid treatment. Existing therapies with neural effects, including anticholinergic bronchodilators, may indirectly influence these pathways. Improved understanding of neuroimmune signalling may therefore help explain treatment variability among horses with SEA and identify novel therapeutic targets.

## 6. Future Directions

A major priority for future research should be the identification and quantification of neuroimmune biomarkers in horses with naturally occurring asthma. Concentrations of SP, NKA, CGRP, NGF, and BDNF should be evaluated in bronchoalveolar lavage fluid (BALF), airway tissue biopsies, and serum samples obtained from healthy horses and horses affected by MEA and SEA. Such studies would provide the first direct evidence regarding neuropeptide and neurotrophin involvement in equine airway inflammation and may help identify biomarkers associated with disease severity and treatment response. Another important research direction concerns the characterisation of sensory nerve receptors within the equine respiratory tract. In particular, expression and distribution of TRP channels, including TRPV1 and TRPA1, should be investigated using immunohistochemistry, in situ hybridisation, transcriptomic approaches, and molecular analyses of bronchial tissue. Given the established role of these receptors in cough hypersensitivity, neurogenic inflammation, and airway hyperresponsiveness in humans and rodents, determining whether similar mechanisms exist in horses represents a critical next step.

The recent demonstration of increased airway innervation in SEA raises important questions regarding neuronal remodelling and airway plasticity. Longitudinal studies should investigate whether airway nerve density changes during disease exacerbation and remission, whether neuronal remodelling correlates with pulmonary function variables and bronchoalveolar lavage cytology, and whether alterations in airway innervation are reversible following environmental modification or anti-inflammatory treatment. Such studies may help determine whether neuronal remodelling is merely a consequence of chronic inflammation or an active contributor to disease progression.

## 7. Conclusions

Current evidence indicates that asthma involves not only inflammatory and immunological dysregulation but also complex neuroimmune interactions that affect airway function and structural remodelling. In humans and rodent models, mechanisms such as sensory nerve activation, tachykinin signalling, neurogenic inflammation, autonomic dysregulation, TRP-channel activation and neuronal remodelling are recognised as important contributors to airway hyperresponsiveness, bronchoconstriction and chronic disease progression.

In horses, environmental exposure to organic dust and microbial particulates remains the principal initiating factor in equine asthma. Nevertheless, the available evidence suggests that neuroimmune pathways may significantly modulate airway dysfunction and contribute to disease persistence. Existing equine studies have demonstrated the presence of neuropeptide-containing nerve fibres in the respiratory tract, increased neurokinin-mediated bronchoconstriction and enhanced airway innervation in severe equine asthma. These findings indicate that neuronal remodelling and neuroimmune signalling may contribute to chronic airway pathology in horses, similar to mechanisms described in human asthma.

Although horse-specific evidence remains very limited, current findings support the hypothesis that neuroimmune dysregulation represents an important component of asthma in horses and may contribute to persistent airway hyperresponsiveness, bronchoconstriction and airway remodelling.

## Data Availability

No new data were created or analyzed in this study. Data sharing is not applicable to this article.

## Abbreviations

The following abbreviations are used in this manuscript:

MEA	Mild-to-moderate equine asthma
SEA	Severe equine asthma
IAD	Inflammatory airways disease
RAO	Recurrent airway obstruction
CGRP	Calcitonin gene-related peptide
TLR	Toll-like receptor
NKA	Neurokinin A
NGF	Nerve growth factor
BDNF	Brain-derived neurotrophic factor
SP	Substance P
